# Case Report: Aplastic anemia related to a novel *CTLA4* variant

**DOI:** 10.3389/fped.2024.1434076

**Published:** 2024-08-16

**Authors:** Geoffrey Hall, Janet G. Markle, James Maiarana, Paul L. Martin, Jennifer A. Rothman, John W. Sleasman, Howard Lederman, Antoine E. Azar, Robert A. Brodsky, Talal Mousallem

**Affiliations:** ^1^ Department of Pediatrics, Division of Allergy and Immunology, Duke University, Durham, NC, United States; ^2^ Department of Pathology, Microbiology and Immunology, and Vanderbilt Center for Immunobiology, Vanderbilt University Medical Center, Nashville, TN, United States; ^3^ Department of Pediatrics, Division of Transplant and Cellular Therapy, Duke University, Durham, NC, United States; ^4^ Department of Pediatrics, Division of Pediatric Hematology/Oncology, Duke University, Durham, NC, United States; ^5^ Department of Pediatrics, Division of Pediatric Allergy, Immunology and Rheumatology, Johns Hopkins, Baltimore, MD, United States; ^6^ Department of Medicine, Division of Allergy and Clinical Immunology, Johns Hopkins, Baltimore, MD, United States; ^7^ Department of Medicine, Division of Hematology, Johns Hopkins, Baltimore, MD, United States

**Keywords:** Aplastic anemia, inborn error of immunity (IEI), novel variant, CTLA-4, haploinsufficiency, hematopoietic stem cell transplantation (HSCT)

## Abstract

A 20-year-old male patient with a history of celiac disease came to medical attention after developing profound fatigue and pancytopenia. Evaluation demonstrated pan-hypogammaglobulinemia. There was no history of significant clinical infections. Bone marrow biopsy confirmed hypocellular marrow consistent with aplastic anemia. Oncologic and hematologic evaluations were unremarkable for iron deficiency, paroxysmal nocturnal hemoglobinuria, myelodysplastic syndromes, T-cell clonality, and leukemia. A next generation genetic sequencing immunodeficiency panel revealed a heterozygous variant of uncertain significance in *CTLA4* c.385T >A, p.Cys129Ser (C129S). Cytotoxic T-lymphocyte-associated protein 4 (CTLA-4) is an inhibitory receptor important in maintaining immunologic homeostasis. To determine the functional significance of the C129S variant, additional testing was pursued to assess for diminished protein expression, as described in other pathogenic *CTLA4* variants. The results demonstrated severely impaired CTLA-4 expression and CD80 transendocytosis, consistent with other variants causing CTLA-4 haploinsufficiency. He was initially treated with IVIG and cyclosporine, and became transfusion independent for few months, but relapsed. Treatment with CTLA-4*-*Ig fusion protein (abatacept) was considered, however the patient opted for definitive therapy through reduced-intensity haploidentical hematopoietic stem cell transplant, which was curative.

## Introduction

Inborn errors of immunity (IEIs) encompass a broad and heterogenous group of genetic disorders which disrupt immunologic homeostasis (1). Traditionally, these conditions have been characterized by which arm(s) of the immune system (e.g., innate, humoral, phagocytic, complement, etc.) are impacted resulting in increased susceptibility to infectious agents. However, coinciding autoimmune/autoinflammatory disease states are increasingly being recognized as a primary or secondary feature among IEIs (2). In particular, immune dysregulation is a primary feature of pathologic variants involving genes responsible for maintaining immunologic homeostasis.

**Figure 1:**
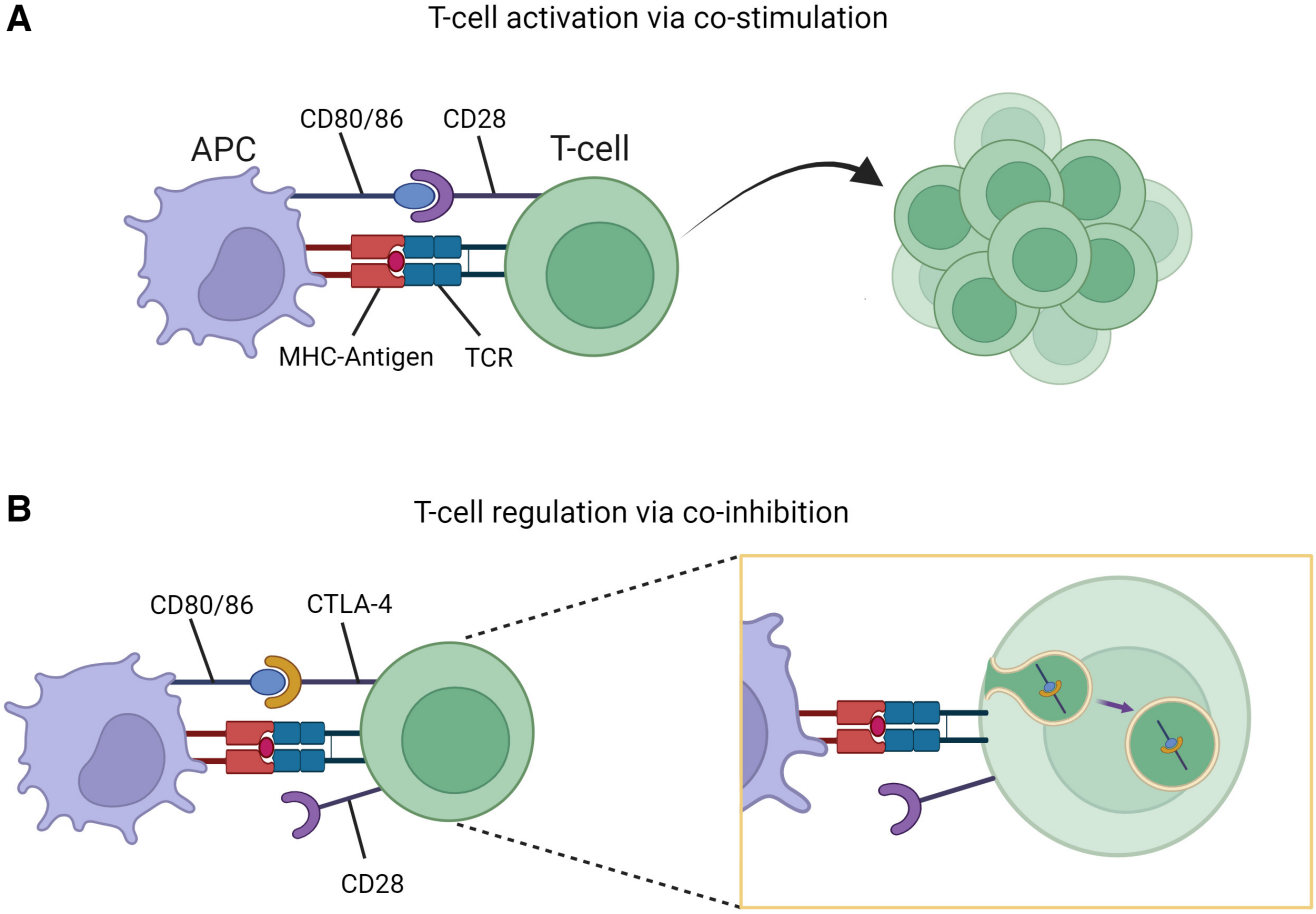
T-cell stimulation and regulation. **(A)** Co-stimulatory signaling through CD80/86-CD28 interaction resulting in T-cell activation and proliferation. **(B)** Co-inhibition through CD80/86-CTLA-4 interaction resulting T-cell regulation due to transendocytosis of CD80/86-CTLA-4 complex. Figure created with Biorender.com.

Cytotoxic T-lymphocyte-associated protein 4 (CTLA-4) is an inhibitory receptor present on T-cells and serves a fundamental role in regulation of immune responses. The process of T-cell (CD4^+^) activation requires both primary and costimulatory signals via interaction with antigen presenting cells (APCs). One such costimulatory signal involves CD28 (present on T-cells) and CD80/86 (present on APCs). Once activated, T-cells upregulate expression of CTLA-4 on their surface which binds to CD80/86 resulting in transendocytosis of the receptors thereby limiting further activation (Figure 1) (3). This coinhibitory mechanism prevents unregulated T-cell activation. Additional immune regulation occurs via T-cell subpopulations such as T-regulatory-cells (T_regs_). These cells constitutively express CTLA-4 providing an additional checkpoint in immune activation (4). In this way, CTLA-4 acts to maintain immunologic homeostasis, with disruptions caused by *CTLA4* variants leading to altered protein expression and clinical disease states.

To our knowledge, no cases of complete CTLA-4 deficiency have been reported in humans. Targeted genetic deletion leading to complete CTLA-4 loss of function in mice leads to fatal multiorgan lymphocytic infiltration primarily due to expansion of unregulated CD4^+^ T-cells (5–8). Complete CTLA-4 deficiency may be incompatible with life in humans. However, pathogenic heterozygous variants in *CTLA4* can lead to disease phenotypes with variable clinical penetrance and expressivity. Phenotypic features reported in patients with CTLA-4 haploinsufficiency include: autoimmune cytopenias, hypogammaglobulinemia, lymphadenopathy, and organ dysfunction (enteropathy, splenomegaly, etc.) from lymphocytic infiltration (9). Additionally, polymorphisms in *CTLA4* have been associated with risk for type 1 diabetes mellitus, Graves' disease, multiple sclerosis, and malignancies (10–13).

In this report, we present a novel *CTLA4* variant manifesting as aplastic anemia and provide functional testing that confirms this novel variant is deleterious to CTLA-4 expression resulting in severely reduced transendocytosis.

## Case description

A 20-year-old male of self-reported White race (ancestry unavailable) presented to medical care for 1 week of persistent and profound incapacitating fatigue. Additional symptoms included palpitations, lightheadedness and exertional dyspnea with ambulation, which all resulted in an inability to participate in collegiate athletics. His medical history was pertinent for celiac disease (confirmed via endoscopic biopsy with symptom resolution after implementing a gluten-free diet), pityriasis alba, and idiopathic wet macular degeneration status-post successful treatment with aflibercept. He did not have a history of other autoimmune disease, immune deficiency or severe/atypical infections. Paternal history was positive for rheumatologic/autoimmune disease including psoriasis, arthritis, and hypothyroidism. Maternal history was unremarkable. There is no history of consanguinity. The remaining family history was pertinent only for thyroid disease of unclear specificity in maternal and paternal grandparents.

Upon presentation, he was anemic (hemoglobin 8.4 mg/dl) and thrombocytopenic (platelet count 18,000/ml). The subsequent day, his white blood cell counts decreased from 4,400 cells/ml to 2,600 cells/ml, consistent with pancytopenia. Iron studies were consistent with mild iron overload and serum copper levels were slightly elevated. He had no prior history of blood transfusions and genetic testing for hemochromatosis demonstrated a heterozygous variant in *HFE* (c.187C >G, p.His63Asp) (H63D). The H63D carrier-status rarely results in clinically significant iron overload (14, 15). The mild elevation in serum copper based on internal lab reference ranges were not thought to be clinically significant and levels up to 158.9 μg/dl are considered normal (16). Additional evaluation including folate, B12, lactate dehydrogenase, and haptoglobin were unremarkable (Table 1). Bone marrow biopsy demonstrated hypocellularity with near absence of erythroid precursors and megakaryocytes, consistent with aplastic anemia. Further hematologic testing was unremarkable including: bone marrow chromosomal analysis, fluorescence *in situ* hybridization for BCR/ABL1 and chromosomal abnormalities (monosomy 5 and 7, trisomy 8, and 20q deletion), myelodysplastic syndrome mutation sequencing, telomere length studies, leukemia flow cytometry immunophenotyping, and T-cell clonality. PNH flow cytometry revealed a loss of GPI-anchored proteins on 0.04% and 0.6% of granulocytes and monocytes, respectively, suggesting that the aplastic anemia may be immune-mediated (18).

**Table 1:** Diagnostic evaluation summary.

Lab	Result	Reference
Presenting labs		
WBC	4,400** **cells/ml	3,200–9,800** **cells/ml
Hemoglobin	**8.4 g/dl**	13.7–17.3 g/dl
Platelets	**18,000/ml**	150,000–450,000/ml
Reticulocyte count (%)	**17,200/ml (0.65)**	28,000–134,000/ml (0.70–2.0)
Folate	11.4 ng/ml	> 6.5 ng/ml
B12	404** **pg/ml	123–730** **pg/ml
Iron	**220 μg/dl**	50–160** **μg/dl
Total Iron binding capacity	**248 μg/dl**	261–478** **μg/dl
Copper	**131 μg/dl**	63–121** **μg/dl
Lactate dehydrogenase	151** **U/L	100–200** **U/L
Haptoglobin	50 mg/dl	30–200 mg/dl
Immunoglobulin M	7 mg/dl	57–273 mg/dl
Immunoglobulin G	122 mg/dl	588–1,573 mg/dl
Immunoglobulin A	12 mg/dl	46–287 mg/dl
Flow cytometry^a^		
WBC	**2,000 cells/ml**	3,200–9,800** **cells/ml
ALC (%)	**268 cells/ml** (13.4)	600–4,200** **cells/ml (10–50)
CD3^+^ (%)	**222 cells/μl (83.0)**	1,543–1,729** **cells/ml (76.1–78.3)^b^
CD4^+^ (%)	**107 cells/μl (40.1)**	942–1,066 (45.7–48.5)^b^
CD8^+^ (%)	**88 cells/μl (32.9)**	544–637 (26.4–28.9)^b^
CD19^+^ (%)	**3 cells/μl (1.3)**	232–281 (11.1–12.8)^b^
NK-cells (%)	**37 cells/μl (13.8)**	188–336 (9.0–10.8)^b^
CD3/CD45RA^+^ (%)	**67 cells/μl** (30.3)	(20.3–44.1)
CD3/CD45RO^+^ (%)	**78 cells/μl** (34.9)	(30.4–51.3)

Abnormal values are bolded.

^a^ Flow cytometry results 2-weeks following presenting labs.

^b^ Reference ranges from Valiathan et al. (17).

Blood count differential demonstrated profound lymphopenia with an absolute lymphocyte count of 268 cells/ml. Lymphocyte enumeration through flow cytometry revealed a T-cell (CD3^+^) count of 222 cells/μl (83%), CD4^+^ count of 107 cells/μl (40.1%), CD8^+^ count of 88 cells/μl (32.9%), naïve T-cell (CD45RA^+^/CD4^+^/CD62l^+^) count of 15 cells/μl (14.4%), B-cell (CD19^+^) count of 3 cells/μl (1.3%), and NK-cell (CD16/56^+^) count of 17 cells/μl (13.8%). T-cell proliferation to phytohemagglutinin (PHA) and tetanus was normal. Immunoglobulin evaluation demonstrated diffuse hypogammaglobulinemia with: IgG (122 mg/dl), IgM (7 mg/dl) and IgA (12 mg/dl). Of note, immunoglobulin levels collected 16-months prior demonstrated a similar pattern with an IgG of 176 mg/dl, an IgM <25 mg/dl, and IgA of 15.7 mg/dl. Antibody titers to tetanus and diphtheria toxoid were protective, and pneumococcal-23 antibody titers were protective to greater than 75% of serotypes tested.

## Diagnostic assessment

A primary immunodeficiency next generation sequencing panel was sent and revealed a novel heterozygous variant of uncertain significance in *CTLA4* (c.385T >A, p.Cys129Ser) (C129S). Paternal testing revealed the same variant. Maternal and sibling testing was negative. Given the patient's clinical phenotype, known risk for immune dysregulation with *CTLA4* variants, and without other identifiable etiologies for the aplastic anemia, a research-based functional assay was pursued.

A CTLA-4 functional assay was performed as previously described (19–21). pCMV6-CTLA4-MycDDK plasmid was obtained from Origene (#RC213631). Construct carrying C129S mutant allele was generated from the wild type (WT) plasmid by site-directed mutagenesis (QuikChange II XL; Agilent Technologies, #200523) according to manufacturer's instructions and validated by Sanger Sequencing. WT or C129S mutant *CTLA4* plasmids were transfected into CHO cells (ATCC # CCL-61) using Lipofectamine 2000 (ThermoFisher Scientific, #11668027), per manufacturer's protocol.

For transendocytosis experiments, transfected CHO cells were co-cultured 1:1 with CellTrace^TM^ Violet (ThermoFisher Scientific #C34557) labelled CHO-CD80^GFP^ cells (A gift of Bodo Grimbacher and David Sansom) for 16 h. CTLA-4 expression and transendocytosis of CD80^GFP^ were measured by flow cytometry on the MACSQuant Analyzer 16 (Miltenyi Biotec). A known loss of function pathogenic variant in *CTLA4*, R51X, was used as a positive control and untransfected CHO cells were used as a negative control. Flow cytometry comparing CTLA-4 expression and CD80 uptake through transendocytosis were run in triplicate.

**Figure 2:**
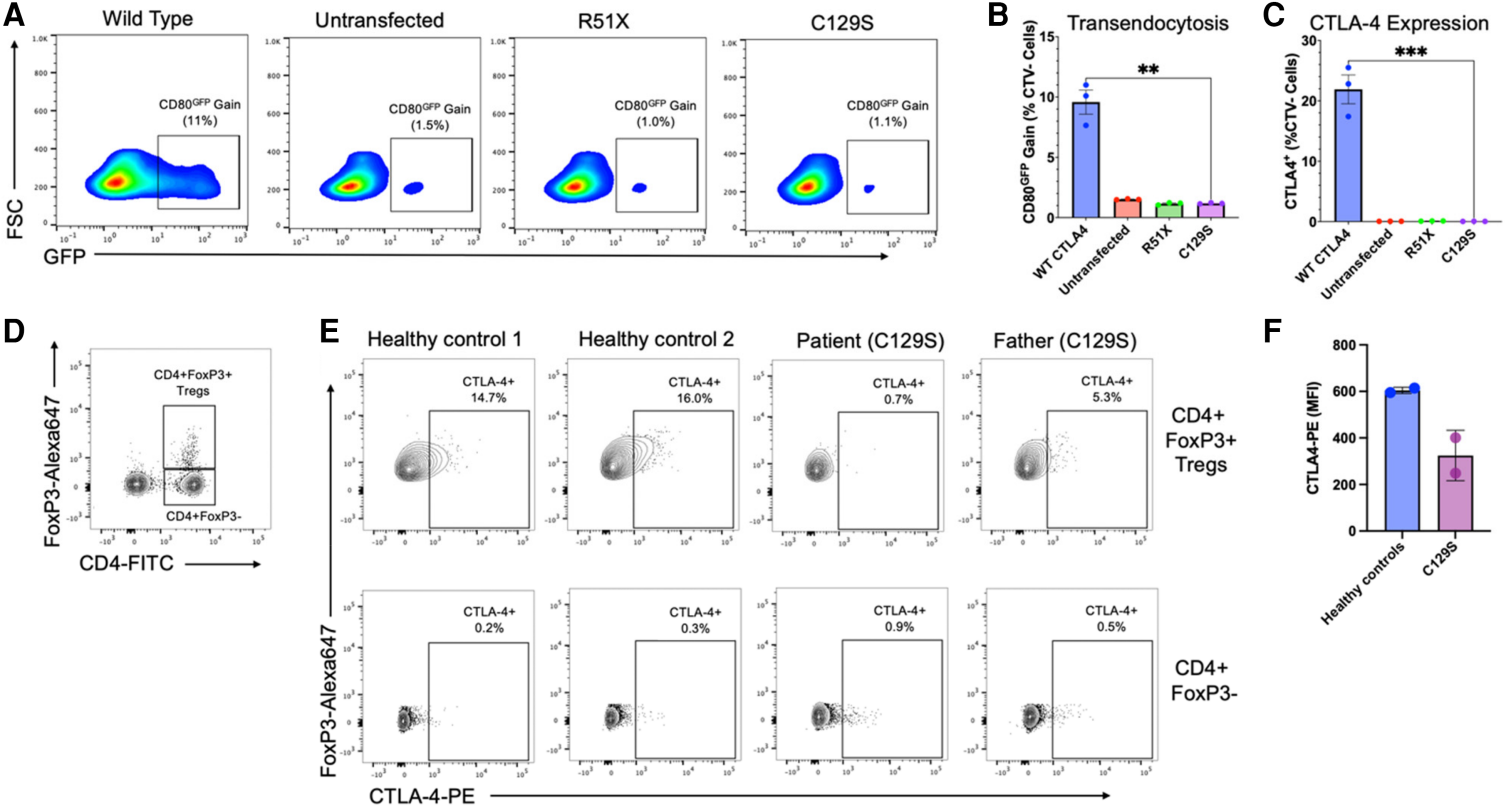
The C129S missense mutation dramatically impairs CTLA-4 expression resulting in decreased transendocytosis. **(A)** Representative example of CD80GFP transendocytosis assay results. These plots show cells previously gated on forward and side scatter profile, and negative for Cell Trace Violet. GFP signal represents percent transendocytosis of CD80. **(B)** Summary results from *n* = 3 independent experiments, performed as in **A**. **(C)** Percent CTLA-4+ CHO cells after transfection with WT or mutant CTLA-4 plasmids (*n* = 3),**p < 0.01, unpaired *t*-tests. **(D)** Flow cytometry analysis of peripheral blood mononuclear cells (PBMCs) to identify CD4 + FoxP3 + regulatory T-cells (T¬¬regs) and CD4 + CD3- cells. **(E)** Analysis of CTLA-4 protein expression by Tregs (upper row) and CD4 + FoxP3 + cells (lower row), gated as shown in **(D)**. **(F)** Mean fluorescence intensity for CTLA-4 on Tregs from two healthy donors, and the patient and his father with the C129S variant.

Results demonstrated that the C129S variant drastically impaired expression of CTLA-4 with a resulting decrease in transendocytosis as compared to wild type. These findings are similar to those seen with the causal allele R51X (Figure 2). Thus, the patient's C129S variant exhibited results consistent with CTLA-4 haploinsufficiency. Moreover, analysis of peripheral blood mononuclear cells (PBMCs) from the patient and his father confirmed reduced CTLA-4 protein levels (Figures 2D,E).

Based on the results from the transendocytosis assay, and the patient's clinical history, the C129S variant was determined to be pathogenic, suggesting that the patient's phenotype was likely due to CTLA-4 haploinsufficiency. Initial therapy consisted of high-dose IVIG (1 g/kg × 2 doses), cyclosporine (maximum dose of 275 mg twice daily), and systemic corticosteroids (maximum dose of 30 mg daily). He responded well to these therapies with notable improvement in red blood cell production seen on bone marrow biopsy. Cyclosporine was then decreased to 175 mg twice weekly with a target trough level of 150–300 ng/ml, and systemic corticosteroids were discontinued. However, 6 months into therapy, he developed renal impairment and was transitioned to 5 mg daily of sirolimus with a trough goal of 5–15 ng/ml, but it was poorly tolerated. In the subsequent 2 months, his aplastic anemia relapsed. Off-label use of abatacept was considered given clinical reports showing positive responses in patients with CTLA-4 haploinsufficiency (22–24). However, given disease severity and the patient's preference, definitive therapy in the form of a haploidentical (sibling, variant negative) hematopoietic stem cell transplantation (HSCT) was pursued (25). Use of haploidentical donor marrow was favored to a matched-unrelated donor for the following reasons: earlier time to transplant, improved total stem cell dose which is critical in non-malignant disease such as aplastic anemia, reduced risk for graft-vs.-host disease due to lower T-cell concentration as compared to peripheral stem cell harvest, and comparable outcomes with use of post-transplant cyclophosphamide. The non-myeloablative conditioning regimen included standard anti-thymocyte globulin (0.5 mg/kg/day on day −9 and 2 mg/kg/day on day −8 and −7), fludarabine (30 mg/m^2^/day on days −6 to −2), cyclophosphamide (14.5 mg/kg/day on days −6 and −5), and total body radiation (400 cGy) on day −1 as previously described (25). Post-transplant course was uncomplicated and graft-vs.-host disease prophylaxis included cyclophosphamide, tacrolimus and mycophenolate mofetil as previously described (25). He achieved >95% CD3^+^ donor chimerism one-month post-transplant with subsequent increase to 100% at around 3-months post-transplant. His most recent chimerism study (1-year post HSCT) continues to show 100% donor chimerism in both peripheral blood and CD3^+^ compartments. He acquired a primary Epstein-Barr infection at around 4-months post-transplantation with mild intermittent clinical symptoms of rash and pharyngitis. He now has resolution of clinical symptoms without intervention, and his most recent EBV DNA is below the threshold of assay detection. Now, 24-months post-transplant, infectious prophylaxes have been discontinued. Due to previous adverse reactions to immunoglobulin replacement, it was not administered post-transplantation. Endogenous immunoglobulin levels normalized by 14-months post-transplant with an IgG of 823 mg/dl, IgM of 69 mg/dl, and IgA of 214 mg/dl. Platelet counts remain appropriate (>150,000/ml) and hemoglobin has been stable (12.5–15.4 g/dl). He remains transfusion independent. Tetanus and diphtheria toxoid titers remain protective. Inactivated vaccinations have been well-tolerated, with plans to administer live vaccines in the future. Growth has been appropriate with a body mass index of 19.7 kg/m^2^. He has resumed normal activities.

## Discussion

CTLA-4 haploinsufficiency is an autosomal dominant condition characterized by reduced CTLA-4 expression and/or function due to variants in *CTLA4* (26). Due to variability in expressivity and penetrance, the condition can be under recognized and underdiagnosed. This was well demonstrated in the case of our patient, where both he and his father shared a common variant in *CTLA4* and exhibited dissimilar phenotypes. While the patient developed severe aplastic anemia, his father has psoriasis, arthritis and hypothyroidism, likely due to CTLA-4 haploinsufficiency as well. In addition, the family history of thyroid dysfunction suggests this variant may be present across generations. Other inborn errors of immunity can also present with similar clinical features to CTLA-4 haploinsufficiency. An example is lipopolysaccharide-responsive beige-like anchor, or LRBA, deficiency. LRBA is a protein responsible for recycling cellular components, including CTLA-4, thereby preventing lysosomal degradation (27). Thus, deficiency of LRBA can lead to reduced CTLA-4 (28).

Therapeutic options for management of immune dysregulatory conditions can be challenging for the practicing immunologist. Immunosuppression to manage autoimmune and autoinflammatory symptoms must be balanced with heightened risk of infection and further marrow suppression. Targeted therapeutic options are limited in management of immune dysregulation, but sometimes can be tailored when the underlying mechanistic pathways are identified. Abatacept is a promising option for patients with CTLA-4 haploinsufficiency. Abatacept is a fusion protein consisting of CTLA-4 fused to the Fc region of human IgG (29). As such, it (at least partially) compensates for the insufficient endogenous expression and/or function of CTLA-4 and binds to CD80/CD86, thereby regulating T-cell stimulation. While abatacept has shown beneficial results in clinical reports, the absence of clinical trials renders it an off-label agent. Moreover, as a replacement therapy, it necessitates life-long treatment in managing CTLA-4 haploinsufficiency, with no available data regarding the long-term clinical implications. In this case, the patient presented with severe clinical disease. Severe or treatment refractory disease including cytopenias and aplastic anemia should prompt an early search for a potential stem cell donor. While abatacept could have been used, the only definitive cure is HSCT. Overall, his post-transplantation outcome has been excellent.

This report outlines a novel *CTLA4* variant with functional confirmation of a pathogenic aberration resulting in CTLA-4 haploinsufficiency. Furthermore, it highlights the diverse phenotypic spectrum increasingly recognized for inborn errors of immunity, especially those involved in immune regulation. Clinical immunologists need to maintain a high index of suspicion when evaluating patients presenting with autoimmunity, autoinflammation, and lymphoproliferation. Genetic sequencing should especially be considered in patients presenting with severe, non-malignant hematologic disease. Establishing a multidisciplinary collaboration is critical to early recognition, management, and occasionally curative intervention for these patients.

## Data Availability

The original contributions presented in the study are included in the article/Supplementary Material, further inquiries can be directed to the corresponding author.
